# Sudden onset of syncope and disseminated intravascular coagulation at 14 weeks of pregnancy: a case report

**DOI:** 10.1186/s12884-020-03083-8

**Published:** 2020-07-14

**Authors:** Mayumi Kamata, Tetsuo Maruyama, Tomizo Nishiguchi, Shinya Iwasaki

**Affiliations:** 1 Department of Obstetrics and Gynecology, Shizuoka City Shimizu Hospital, 1231 Miyakami, Shimizu-ku, Shizuoka-shi, Shizuoka, 424-8636 Japan; 2 Department of Obstetrics and Gynecology, Keio University School of Medicine, 35 Shinanomachi, Shinju-ku, Tokyo, 160-8582 Japan; 3 Department of Obstetrics, Perinatal Medical Center, Shizuoka Children’s Hospital, 860, Urushiyama, Aoi-ku, Shizuoka-shi, Shizuoka, 420-8660 Japan

**Keywords:** Amniotic fluid embolism, Syncope, Disseminated intravascular coagulation, Subchorionic hematoma, Case report

## Abstract

**Background:**

Amniotic fluid embolism (AFE), also known as anaphylactoid syndrome of pregnancy (ASP), typically occurs during labor and may result in cardiorespiratory collapse and disseminated intravascular coagulation (DIC). There are reports describing less typical presentations of AFE/ASP in which patients do not necessarily have the classic triad of hypoxia, hypotension, and coagulopathy. AFE/ASP rarely occurs in the absence of labor, but such cases may involve medical or surgical abortion, spontaneous miscarriage, or obstetrical procedures including amniocentesis and amnioinfusion. There are, however, no previously reported cases of AFE/ASP with sudden loss of consciousness and disseminated intravascular coagulation occurring during early pregnancy, in the absence of any intervention or obstetric event.

**Case presentation:**

A 32-year-old G3P2 Japanese woman had sudden-onset syncope at 14 weeks’ gestation. On arrival at our hospital, her level of consciousness was severely disturbed as determined by the Glasgow Coma Scale. Although her vital signs were initially stable, blood samples collected intravenously and by femoral artery puncture did not coagulate. A subchorionic hematoma with active extravasation of blood was apparent on contrast-enhanced computed tomography. Two hours after her arrival, she developed hypovolemic shock with progression of DIC, presumably due to intrauterine and retroperitoneal bleeding. After transfusion of blood products; treatments for DIC including the use of recombinant human soluble thrombomodulin, ulinastatin, and corticosteroids; and hysterectomy, her level of consciousness and physical condition improved remarkably. Later investigation of preoperative blood samples revealed that serum levels of AFE/ASP-associated markers were elevated. Immunohistochemical studies on the excised, unruptured uterus showed that amniotic fluid components were present inside a uterine blood vessel.

**Conclusions:**

This is the first reported patient with sudden-onset syncope and DIC, but without apparent cardiorespiratory collapse, with the highly likely etiology of AFE/ASP occurring at the beginning of the second trimester of pregnancy and in the absence of intervention or delivery. Maternal collapse with DIC during any stage of pregnancy should be considered an AFE/ASP-associated event, even in the absence of labor or obstetric procedures. This event may occur in the presence of subchorionic hematoma alone.

## Background

Amniotic fluid embolism (AFE) occurs when amniotic fluid enters the bloodstream of the gravid mother, usually during labor, triggering a serious and rapid reaction that can result in cardiorespiratory collapse and disseminated intravascular coagulation (DIC) [1]. Also known as anaphylactoid syndrome of pregnancy (ASP) [2–5], AFE accompanies anaphylaxis-like symptoms of shock. These symptoms cannot be explained by simple occlusion of the mother’s pulmonary microvasculature by amniotic fluid components.

A rare but devastating condition, AFE/ASP occurs in approximately 1 in 40,000 deliveries, with a reported mortality ranging from 20 to 60% [1]. Reportedly, 70% of AFE/ASP occurs during vaginal delivery, 11% after delivery, and 19% during cesarean delivery [2]. There are reports of AFE/ASP occurring in the second trimester during induced abortion [6, 7]. These events rarely occur in the absence of labor, but there are reports of AFE/ASP occurring during medical or surgical abortion [6–8], spontaneous miscarriage [9], or obstetric procedures including amniocentesis [10] and amnioinfusion [11]. To the best of our knowledge, there are no reports of AFE/ASP occurring during the course of a viable pregnancy without intervention or delivery.

We present a patient who very likely had AFE/ASP manifesting with sudden-onset syncope and DIC but without apparent cardiorespiratory collapse. The patient was in early pregnancy, and there was no intervention or delivery to precipitate the event.

## Case presentation

**Fig. 1:**
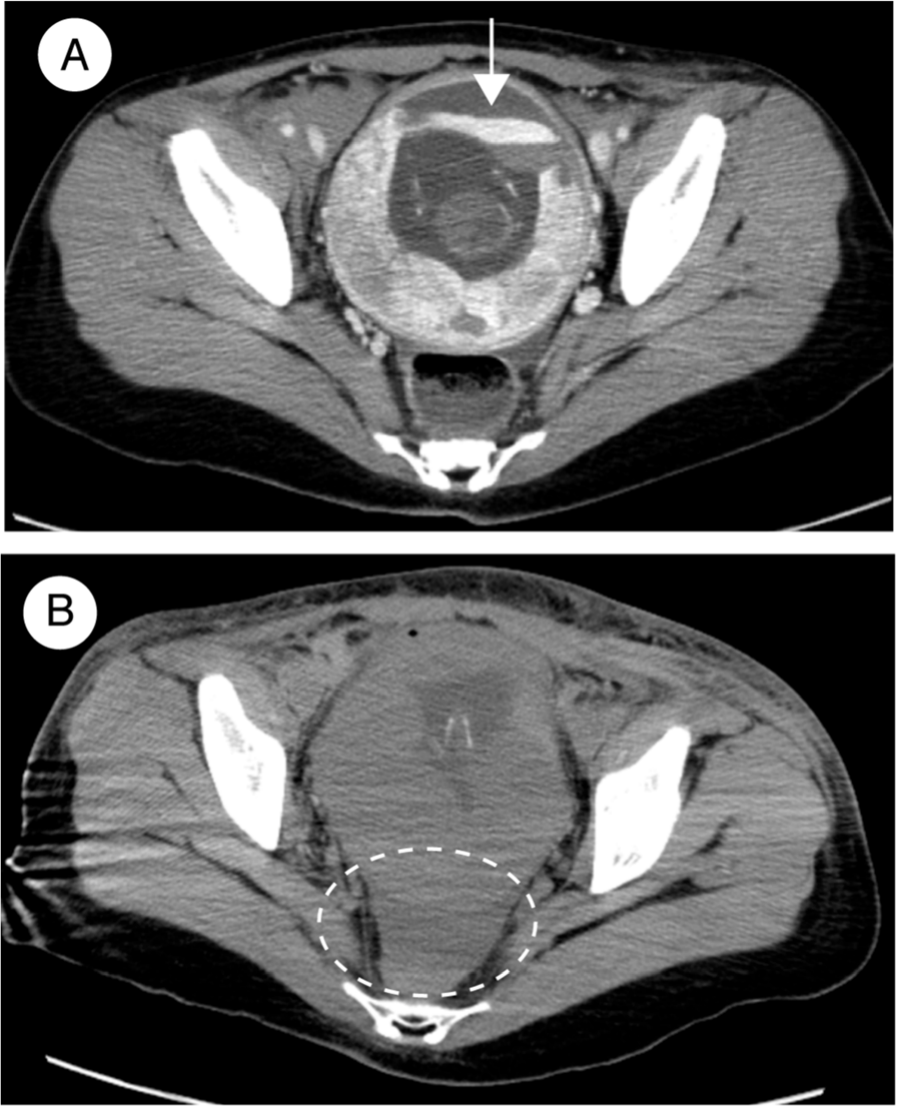
Axial pelvic computed tomography (CT) images at 1 h **a** and 11 h **b** after presentation. **a** Contrast-enhanced CT demonstrating active extravasation into a subchorionic hemorrhage (arrow). **b** Noncontrast CT demonstrating a retroperitoneal hematoma in the pelvis (dotted circle)

A 32-year-old G3P2 woman at 14 weeks’ gestation was brought to the emergency department by ambulance for sudden-onset syncope. She had 2 previous cesarean deliveries. The patient and her husband were elementary school teachers with 2 children, aged 5 and 3 years. The patient had a small ventricular septal defect that did not require treatment. She had no history of other past illness, including seizures or syncopal episodes, and she took no medication. On the day of her syncopal episode, she consumed the same evening meal as her husband and 2 children. She then took a bath. The episode occurred suddenly, while she was putting her children to bed and was actually in bed with them. Her 5-year-old child immediately called his father, who called an ambulance. On arrival to the emergency department, her Glasgow Coma Scale score was 6/15 (E1V2M3), and she was uttering strange sounds and exhibiting restlessness, with tonic extension of both arms. We treated her in accordance with the Immediate Cardiac Life Support protocol, developed by the Japanese Association for Acute Medicine and launched in 2002 as a part of the introductory training course for cardiopulmonary resuscitation [12]. Both her pupils were 6 mm in diameter and nonreactive to light. She had no history of ingestion or exposure and no clinical signs associated with poisoning. We therefore did not perform toxicology screening. Her initial vital signs were: blood pressure, 137/65 mmHg; pulse, 97 beats/min; body temperature, 36.7 °C; and oxygen saturation, 95% on 10 L/min oxygen administered by face mask. Her oxygen saturation on room air was unknown because she arrived at the hospital with a face mask in place. There were no other cardiopulmonary resuscitation interventions performed before her arrival at our hospital. Blood samples were collected intravenously and by femoral artery puncture, and a complete blood count, biochemistry profiling, and blood gas analysis were performed. Her hemoglobin level was 13.5 g/dL, and her platelet count was 150, 000/μL. The blood glucose concentration was 143 mg/dL, and her liver enzymes were slightly elevated, with an aspartate aminotransferase level of 72 IU/L and an alanine aminotransferase level of 15 IU/L. Her serum pH was 7.353, blood lactate was 6.1 mmol/L, and arterial partial pressure of oxygen was 173 mmHg. As her blood samples did not coagulate, neither prothrombin time nor activated partial thromboplastin time was measurable, suggesting a deficiency of coagulation factors. She had no genital bleeding, but transabdominal ultrasonography revealed a subchorionic hematoma (SCH), approximately 8 × 3 cm. This had been observed 1 week prior and was essentially unchanged in size. The fetus was living. Computed tomography (CT) and magnetic resonance imaging of the head and thoracoabdominal contrast-enhanced CT revealed no evidence of pulmonary embolism, venous thrombosis, or intraperitoneal bleeding. Extravasation of contrast into the SCH was observed (Fig. 1a).

Two hours after her arrival, the patient exhibited a drop in blood pressure to 75/49 mmHg together with a decrease in her Hgb level, from 9.8 g/dL to 6.3 g/dL, and a decrease in her platelet count, from 94,000/μL to 70,000/μL. We started transfusing packed red blood cells (PRBC), fresh frozen plasma (FFP), and concentrated platelets under the presumed diagnosis of DIC. She gradually regained consciousness and ultimately responded to her name. The DIC resolved, and her fibrinogen level increased, from unmeasurable at 2 h after arrival to 36 mg/dL at 5 h and 109 mg/dL at 8 h. Despite these gradual improvements, her blood pressure decreased again and her condition became unstable. Pelvic CT performed 11 h after arrival revealed an increase in the size of the SCH and a large retroperitoneal hematoma (Fig. 1b).

We suspected that the intrauterine hemorrhage and retroperitoneal bleeding, presumably originating from the uterus and the femoral artery puncture site, respectively, might worsen her general condition and coagulation status. We provided additional blood-component transfusions and subsequently preformed a supracervical hysterectomy. The ratio of the preoperative transfused products, PRBC: FFP: platelets, was 1:1.4:1. We observed a massive retroperitoneal hematoma and noted that the uterus was larger than expected but not ruptured, indicating that the retroperitoneal bleeding did not originate from the uterus. The amount of intraoperative blood loss was approximately 2400 g. On postoperative day 6, she had lower abdominal pain with a fever of 37.7 °C and an intrapelvic hematoma was detected by CT; infection was suspected. Drainage and flushing of the hematoma with antibiotic treatment improved her condition. She was discharged from the hospital 22 days after the hysterectomy. She is now back to her job as an elementary school teacher. She has had no sequelae and requires no medication.

**Fig. 2:**
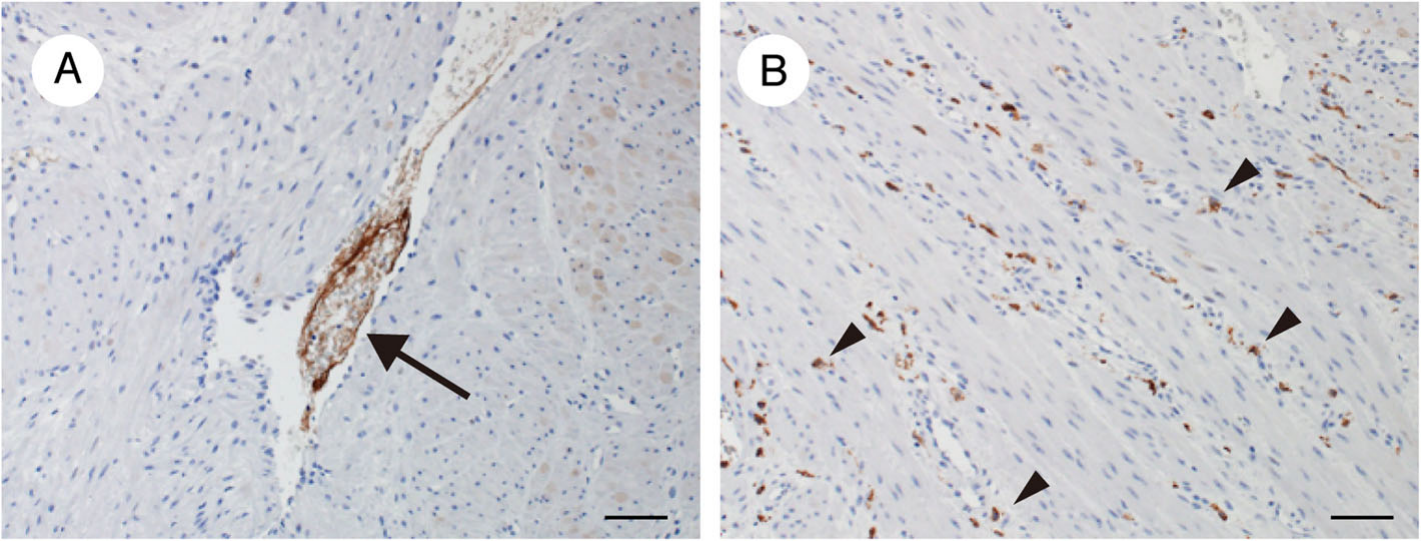
Immunohistochemical staining of the myometrium (hysterectomy specimen). **a** Complement component 5a receptor-positive cells (arrowheads) are present in the myometrium. **b** Zinc coproporphyrin-1-positive material (arrow) in a uterine vessel. Bars, 100 μm

Considering our patient’s clinical course and laboratory and imaging data, we felt that AFE was the most likely cause, despite the absence of severe hypoxia, because AFE provokes both DIC and maternal collapse resulting in reduced or absent consciousness [13, 14]. We therefore performed a detailed pathologic examination of the hysterectomy specimen, revealing an edematous myometrium. The presence of amniotic fluid inside a uterine blood vessel was evidenced by positive staining for zinc coproporphyrin-1 (Zn-CP1), which is reactive with fetal and amniotic components [15] (Fig. 2a). In addition, many complement component 5a (C5a) receptor-positive cells were present in the myometrium (Fig. 2b), suggesting the presence of an anaphylactoid reaction [15]. Serum levels of Sialyl Thomsen-nouveau antigen (STN), specific for amniotic fluid, were elevated to 280.0 U/mL (reference value, < 45.0 U/mL), and her interleukin 8 (IL-8) level was elevated to 494.0 pg/mL (reference value, < 2.0 pg/mL). These findings collectively and strongly suggested the presence of AFE, in particular DIC-type AFE [15].

## Discussion and conclusions

Our patient initially exhibited sudden-onset collapse with DIC. The term “collapse” refers to a range of medical conditions, from simple syncope to a catastrophic cardiac event heralding imminent death [13]. Its causes include hemorrhage, thromboembolism, AFE/ASP, cardiac disease, sepsis, drug toxicity or overdose, eclampsia, intracranial hemorrhage, and anaphylaxis [14]. The onset of DIC during pregnancy is caused by preeclampsia; eclampsia; hemolysis, elevated liver enzymes, low platelet count (HELLP) syndrome; acute fatty liver; placental abruption; postpartum hemorrhage; AFE/ASP; and sepsis [16]. Because AFE/ASP is capable of provoking both maternal collapse and DIC, we speculated that AFE/ASP might be the most likely cause for this patient’s presentation, despite the absence of severe hypoxia.

A rare obstetric emergency, AFE/ASP occurs when amniotic fluid enters the maternal bloodstream, rapidly triggering cardiorespiratory collapse and DIC [1]. Kanayama et al. analyzed more than 400 patients with AFE and reported that there are 2 etiologies [15]. First, fetal materials may create physical obstruction in the maternal microvessels in various organs, including the lung. Second, the amniotic liquid itself may cause an anaphylactoid reaction that leads to cardiopulmonary or cerebral vasospasm and activation of platelets, white blood cells, and complement. The former type of AFE/ASP is characterized mainly by cardiopulmonary collapse, whereas the latter, termed DIC-type AFE, involves the presence of DIC and atonic bleeding [15]. The DIC type of AFE is conceptually similar to ASP [2, 3] because both implicate the anaphylactoid reaction to amniotic fluid components as the cause of maternal collapse with coagulopathy [2, 3, 15]. The serum markers Zn-CP1; STN; complement C3, C4, C5a; and IL-8 have been used as indicators for AFE/ASP [15].

Our patient had amniotic fluid present inside a uterine blood vessel, as evidenced by positive staining for Zn-CP1 (Fig. 2a). The simple presence of amniotic fluid and fetal components in pathology specimens does not definitely indicate AFE/ASP, because amniotic fluid is occasionally identified in the central circulation of normal pregnant patients and of patients with complications other than AFE/ASP [17]. We also cannot completely eliminate the possibility that the operative procedure itself could have created these findings in the uterus, although we handled the uterus gently and did not incise the uterine body to enter the cavity during hysterectomy. Notably, our patient also had an edematous myometrium containing many C5a receptor-positive cells (Fig. 2b), suggesting an anaphylactoid reaction. Taken together, it is possible that the entry of amniotic fluid components into the uterine blood vessel might have induced an anaphylactoid reaction both locally and systemically, involving the coagulation system and the brain and resulting in DIC and loss of consciousness through cerebral vasospasm. Furthermore, our patient had elevated levels of serum STN, specific for amniotic fluid, and serum IL-8, an inflammatory cytokine indicative of systemic inflammatory response syndrome (*SIRS*). As with SIRS, activation of proinflammatory mediators is thought to be involved in AFE [1]. These findings collectively suggested the presence of AFE, specifically DIC-type AFE [15].

Although there is a lack of international consensus regarding the diagnostic criteria for AFE/ASP [15, 17], Clark et al. proposed 4 uniform criteria for research studies [17]: the sudden onset of cardiorespiratory arrest or hypotension with respiratory compromise; documentation of overt DIC; clinical onset during labor or within 30 min of delivery of the placenta; and the absence of fever during labor [17]. Based on these criteria, only classic and typical presentations are recognized as true AFE/ASP. Because our patient did not satisfy the first or third criterion, it is possible that an etiology other than AFE/ASP was responsible for the sudden onset of syncope and DIC. As we did not perform any drug or toxicology screening, we cannot completely exclude the possibility that her presentation was attributable to poisoning or medication. Less typical presentations of AFE/ASP are classified as the following nonspecific medical conditions, each much more common than AFE/ASP: hemorrhage, sepsis, anesthetic accident, pulmonary thromboembolism, or systemic anaphylaxis [17]. However, based on our patient’s clinical and laboratory findings, these other medical conditions [16, 17] are not likely to account for her initial condition, with sudden-onset syncope and DIC. Furthermore, the details of the onset of syncope with DIC; the absence of apparent clinical signs associated with poisoning; the absence of a significant medical history, including seizures or syncopal episodes; and the absence of medication use, collectively indicate a low possibility of poisoning or medication as the etiology of her presentation.

Only less typical types of AFE/ASP, in particular DIC-type AFE [15], seem to be consistent with her initial condition. She had DIC; unconsciousness, presumably due to AFE/ASP-induced cerebral vasospasm; amniotic fluid components inside a uterine blood vessel; many anaphylactoid reaction-associated C5a receptor-positive cells in the myometrium; an elevated serum amniotic fluid-specific antigen level; and elevated serum levels of inflammatory cytokines, indicative of SIRS and possibly involved in AFE. We therefore propose the following possible mechanism to explain her presentation. First, amniotic fluid or fetal components entered the maternal blood stream, presumably from the fetomaternal interface at the pre-existing SCH that caused a breach in the physiologic barrier between fetus and mother. These materials provoked cerebral vasospasm and affected the coagulation system through an anaphylactoid reaction, resulting in sudden-onset syncope and DIC. It is possible for AFE to occur without labor, for instance, following medical and surgical abortion [6–8], spontaneous miscarriage [9], or obstetric procedures including diagnostic amniocentesis [10] and amnioinfusion [11]. The absence of apparent cardiorespiratory collapse in our patient indicates that DIC-type AFE may have occurred rather than conventional AFE. Subsequent DIC-associated bleeding at the pre-existing SCH and, presumably, at the femoral artery puncture site led to enlargement of the SCH and a massive retroperitoneal hematoma, respectively. There is a previous report of a massive retroperitoneal hematoma occurring secondary to a femoral artery puncture [18], although we could not find any bleeding points in either femoral artery during our patient’s surgery. Retroperitoneal and intrauterine massive bleeding worsened her DIC and enhanced further blood loss, resulting in a hemodynamic instability. Removal of the affected uterus; blood-component transfusions; and anti-DIC treatments, including the use of recombinant human soluble thrombomodulin, ulinastatin, and corticosteroids, led to immediate improvement.

This is the first reported patient with sudden-onset syncope and DIC, without apparent cardiorespiratory collapse, in whom the etiology is highly likely to be AFE/ASP at the beginning of the second trimester of pregnancy, in the absence of intervention or delivery. Subsequent worsening of out patient’s intrapelvic hemorrhage complicated her clinical course, but we emphasize that the sudden onset of maternal collapse with coagulopathy during any stage of pregnancy should be considered as an AFE/ASP-associated event. It may be very rare, but it is possible that SCH may allow amniotic fluid components to enter the maternal circulation and thereby provoke AFE/ASP during early pregnancy.

## Data Availability

The data were obtained from the patient’s medical record and are not publicly available.

## Abbreviations

AFE: Amniotic fluid embolism
ASP: Anaphylactoid syndrome of pregnancy
C: Complement
CT: Computed tomography
DIC: Disseminated intravascular coagulation
IL-8: Interleukin 8
FFP: Fresh frozen plasma
HELLP: Hemolysis, elevated liver enzymes, low platelet count
PRBC: Packed red blood cells
SCH: Subchorionic hemorrhage
SIRS: Systemic inflammatory response syndrome
STN: Sialyl Thomsen-nouveau antigen
Zn-CP1: Zinc coproporphyrin-1
