# Correction to: Sudden onset of syncope and disseminated intravascular coagulation at 14 weeks of pregnancy: a case report

**DOI:** 10.1186/s12884-020-03125-1

**Published:** 2020-07-28

**Authors:** Mayumi Kamata, Tetsuo Maruyama, Tomizo Nishiguchi, Shinya Iwasaki

**Affiliations:** 1 Department of Obstetrics and Gynecology, Shizuoka City Shimizu Hospital, 1231 Miyakami, Shimizu-ku, Shizuoka-shi, Shizuoka, 424-8636 Japan; 2 Department of Obstetrics and Gynecology, Keio University School of Medicine, 35 Shinanomachi, Shinju-ku, Tokyo, 160-8582 Japan; 3 Department of Obstetrics, Perinatal Medical Center, Shizuoka Children’s Hospital, 860, Urushiyama, Aoi-ku, Shizuoka-shi, Shizuoka, 420-8660 Japan

**Correction to: BMC Pregnancy and Childbirth 20, 406 (2020)**

**https://doi.org/10.1186/s12884-020-03083-8**

Following publication of the original article [1], the authors identified an error in Fig. 2 legend. The legend of Fig. 2a and Fig. 2b was reversed.

**Fig. 2:**
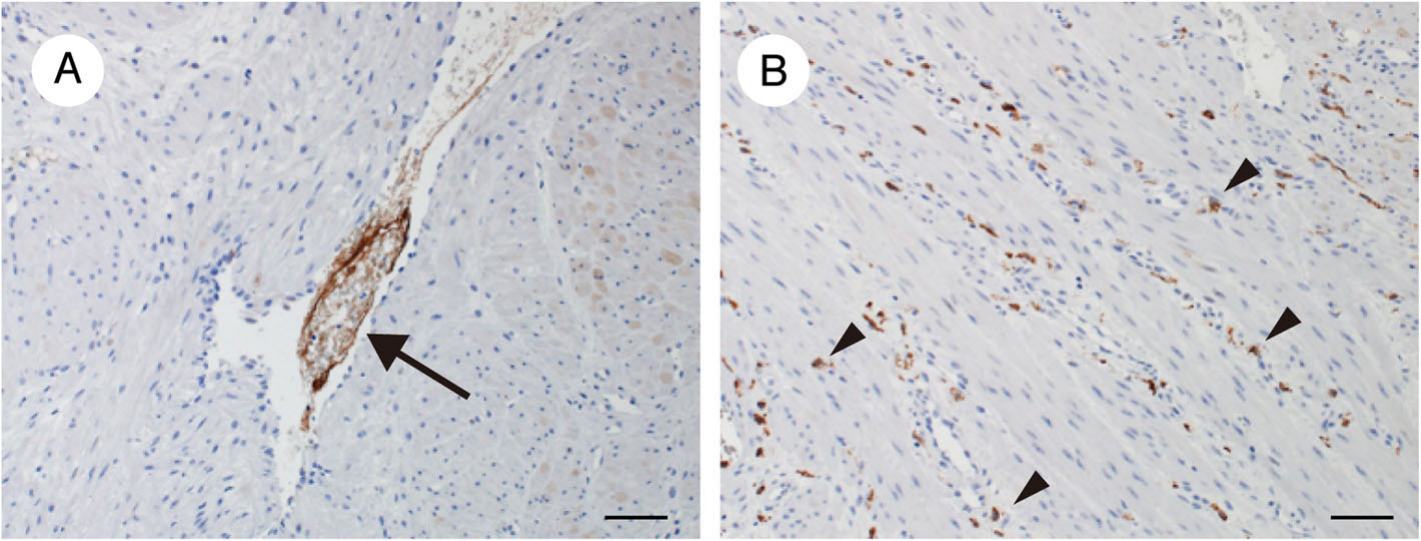
Immunohistochemical staining of the myometrium (hysterectomy specimen). **a** Zinc coproporphyrin-1-positive material (arrow) in a uterine vessel. **b** Complement component 5a receptor-positive cells (arrowheads) are present in the myometrium

The figure and corrected legend has been included in this correction and the original article has been corrected.
